# Stress and fitness in parthenogens: is dormancy a key feature for bdelloid rotifers?

**DOI:** 10.1186/1471-2148-7-S2-S9

**Published:** 2007-08-16

**Authors:** Claudia Ricci, Manuela Caprioli, Diego Fontaneto

**Affiliations:** 1 Dipartimento di Biologia, Università degli Studi di Milano, 20133 Milano, Italy; 2 Current address: Imperial College London, Division of Biology, Silwood Park Campus, Ascot, Berkshire, SL5 7PY, UK

## Abstract

**Background:**

Bdelloid rotifers are the most common and abundant group of animals that reproduce by ameiotic parthenogenesis, only. They are common in temporally ephemeral habitats, and it is unclear if they dwell in unstable habitats because are excluded from better conditions by stronger competitors, or because they need unstable conditions for their success. We tested the hypothesis that bdelloids 'require' stressful conditions for their persistence by comparing fitness-related traits of stressed (desiccated, D) and unstressed (hydrated, H) lines of two species, *Adineta ricciae *and *Macrotrachela quadricornifera*.

**Results:**

For both bdelloid species, fecundity was significantly lower in H than in parallel D line. Fitness components decreased with time progressively in the H line but not in the D line. Recovery rates of D lines were recorded after every desiccation and did not reveal any trend in time, suggesting that no selection was operating.

**Conclusion:**

Stress in the form of reiterated desiccations seemed to help both bdelloid species to keep fitness stable; in contrast under stable conditions, like permanent hydration, these bdelloid species had poorer performances. Bdelloids, although aquatic animals, are not only efficient in tolerating desiccation, but seem somehow dependent on anhydrobiosis, a circumstance that might represent a key event in their life cycle. If this is true, life in unpredictable habitats should not be seen as the result of competitive exclusion from 'easier' habitats, but a requirement for long-term survival of these parthenogenetic animals.

## Background

Nearly all kinds of eukaryotic organisms reproduce sexually by joining the two processes of meiosis and karyogamy: genes from two parents are combined and produce genetically different organisms. A few scattered groups of organisms are able to produce by mitosis, rather than meiosis, female gametes which will develop into offspring that are genetically identical to their mothers. This process is called parthenogenesis and provides faithful replication of the genome, barring mutation, and high reproductive efficiency. Although parthenogens should have a reproductive advantage over their sexual relatives, they seem to be unsuccessful over the long term and to have short evolutionary lifespans [1,2]. Nevertheless, parthenogenetic species are distributed in many geographical areas where tend to occupy habitats characterized by unstable conditions [3]. One possible interpretation for this is that disturbed areas provide refuges where the contact with bisexual species is prevented, and this favours the settlement and establishment of parthenogenetic species. Alternatively, the preferential occupancy of disturbed areas might be because the parthenogens benefit from recurrent environmental stress. Their abundance in catastrophic or disturbed habitats can be easily attributed to their ability of colonization, but, under the continuous struggle between competitors, predators and parasites, parthenogens are expected to be doomed.

Bdelloid rotifers constitute the largest, oldest, most diverse animal taxon for which there is morphological, cytological, and molecular evidence for long-term parthenogenesis [4-9]. The group has likely evolved after having acquired the ameiotic reproduction as supported by recent results on the evolution of bdelloid genomes [e.g. [5,6]]. In spite of its conservative reproductive modality, taxon Bdelloidea has been able to evolve and differentiate into a few hundreds of distinguishable morphologies, traditionally considered species [10], which occupy a variety of freshwater habitats in all continents [11].

The sediments of lotic and lentic waters, as well as the thin water film around soil particles, mosses and lichens are bdelloids' common habitats, where temperature, food availability, chemical conditions and water content change quickly and unpredictably. The instability and uncertainty seem to favour bdelloid presence, as about 90% of the known species (about 400) occur in 'terrestrial' habitats [12]. In response to environmental stress, like desiccation or starvation, bdelloids enter dormancy and at recovery do not show either decreased fecundity or early death [13,14]. Recent work indicates that, for some species, mothers that have been through desiccation produce daughters of increased fitness and longevity, suggesting the existence of some repair processes associated with recovery from desiccation which may have a beneficial effect beyond surviving desiccation [15]. Some indirect experimental evidence seem to point to the same direction; a part of a clonal culture of a bdelloid species, *Philodina roseola*, was maintained under constantly hydrated conditions, and another part was desiccated. After recovery, both lines were checked and those recovered were found to have higher fertility [11].

It has been suggested that the unusual ecology of bdelloids, that through dormancy can cause frequent DNA damage and repair, may have facilitated adaptations that favoured their long term evolutionary survival in absence of sexual reproduction and of recombination [16]. If this is true, a bdelloid population should be predicted to have a higher fitness under severe stress than a parallel 'unstressed' line. We intend to test this hypothesis by comparing the life-history traits of two lines, one stressed repeatedly and another continuously unstressed, of two bdelloid species, *Macrotrachela quadricornifera *Milne, 1886, family Philodinidae, (called *M.q*.), and *Adineta ricciae *Segers & Shiel, 2005, family Adinetidae, (called *A.r*.). Both species live in habitats where desiccation occurs, are naturally capable of anhydrobiosis, a form of dormancy induced by water loss, and at re-hydration appear to ignore the time spent dry [15]. *A.r*. was originally collected from the dry sediments of an Australian billabong, at re-hydration recovers in high percentages and seems to increase its fecundity when compared to a parallel hydrated control [15,17,18]. *M.q*. was isolated from a moss around a spring-fed pond in Northern Italy, is cultivated in the lab since several years and after recovery has same fecundity as its hydrated control [13,19].

From a laboratory clonal culture of either species two subpopulations were isolated; one was maintained under continuous hydration for 15 months ("hydrated line" H), and the other one was desiccated for 7 days at monthly intervals ("desiccated line" D). For the D line of both species recovery percentages were assessed at each re-hydration. The 15 month experiment was repeated in two different years. The effect of desiccation on population fitness was assessed by running life-table experiments on cohorts established from the ancestor population (M0, used for reference) and from the H and D lines after four (M4), eight (M8), and twelve (M12) subsequent desiccations, that correspond to 4, 8, and 12 months respectively.

## Results

### *Adineta ricciae*

Recovery percentages recorded after each of the 15 desiccation events varied between 88% and 97% in the first experiment, and between 86% and 94% in the second one (Additional file 1). A two-way ANOVA revealed that neither the number of desiccations (F_15,82 _= 1.26, P = 0.243) nor the replicate experiments (ANOVA test: F_1,81 _= 0.04, P = 0.84) affected the recovery of the D population.

During the whole lifespan each bdelloid of the D line produced 26–28 eggs in a mean 9.5 reproductive days (Table 1). The fecundity of their ancestors that represented the reference cohort (M0) was not different, while the eggs produced by the H line were a mean of 17 eggs after 8–12 months of stable hydration. The reproductive effort of the D line ranged from 2.9 to 3.2 eggs/female x day after 12 desiccation events (M12), while in H line the reproductive effort was 1.3 after 12 months of continuous hydration.

**Table 1:** Life-history parameters.

*Adineta ricciae*						
	fecundity	reproductive days	reproductive effort	eggs produced till 10-d old	age at first reproduction	longevity
M0	28.51 (±0.45)	14.79 (±0.60)	2.03 (±0.07)	24.19 (±0.34)	2.07 (±0.07)	36.03 (±1.82)
M4 H	22.75 (±0.6)	7.14 (±0.2)	3.19 (±0.0)	22.46 (±0.5)	2.18 (±0.1)	30.61 (±1.85)
M4 D	25.85 (±0.84)	8.52 (±0.44)	3.16 (±0.12)	24.93 (±0.7)	1.96 (±0.06)	34.40 (±2.86)
M8 H	16.65 (±1.18)	9.08 (±0.56)	1.85 (±0.10)	15.54 (±1.05)	2.27 (±0.09)	18.27 (±1.24)
M8 D	26.06 (±1.24)	8.50 (±0.41)	3.14 (±0.17)	25.46 (±1.20)	1.71 (±0.09)	23.64 (±2.13)
M12 H	17.30 (±0.54)	14.30 (±0.58)	1.28 (±0.08)	11.78 (±0.76)	3.19 (±0.14)	38.52 (±2.30)
M12 D	28.15 (±0.70)	10.11 (±0.46)	2.92 (±0.12)	26.78 (±0.86)	2.07 (±0.09)	27.85 (±2.41)

*Macrotrachela quadricornifera*						
	fecundity	reproductive days	reproductive effort	eggs produced till 10-d old	age at first reproduction	longevity

M0	26.81 (±0.62)	27.02 (±1.00)	1.05 (±0.04)	10.84 (±0.18)	3.58 (±0.08)	52.28 (±2.12)
M4 H	19.96 (±0.93)	25.36 (±1.41)	0.82 (±0.04)	9.46 (±0.34)	2.93 (±0.07)	46.43 (±3.44)
M4 D	26.07 (±0.72)	22.50 (±0.89)	1.20 (±0.05)	14.32 (±0.49)	2.82 (±0.07)	39.36 (±2.43)
M8 H	14.78 (±0.94)	19.81 (±1.51)	0.78 (±0.03)	7.74 (±0.39)	3.78 (±0.13)	38.86 (±3.28)
M8 D	24.81 (±1.17)	20.81 (±0.87)	1.24 (±0.07)	13.22 (±0.63)	2.70 (±0.10)	41.61 (±3.13)
M12 H	21.39 (±0.73)	28.96 (±1.22)	0.76 (±0.02)	9.96 (±0.40)	3.21 (±0.08)	52.61 (±2.80)
M12 D	27.04 (±0.87)	21.68 (±1.20)	1.34 (±0.08)	17.25 (±0.51)	2.96 (±0.04)	38.96 (±2.52)

Mean values (± standard error) of hydrated (H) and desiccated (D) lines of *A. ricciae *and *M. quadricornifera *at start of the experiment (M0) and after four (M4), eight (M8) and twelve (M12) months (= months of continuous hydration for H or subsequent desiccations for D). Fecundity, reproductive days, reproductive effort (= number of eggs per reproductive day), eggs produced till 10-d-old, age at the first reproduction, and longevity are reported.

**Figure 1:**
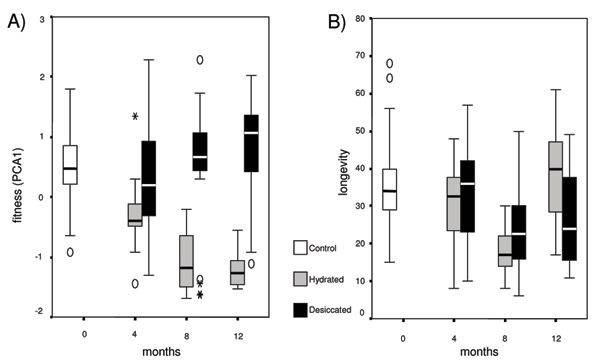
***Adineta ricciae*, life history traits**. Box-plots of fitness components (A) and longevity (B) at start (control, white) and after 4, 8, 12 months of hydration (grey) and subsequent desiccations (black). The box represents the interquartile range which contains the 50% of values. The whiskers are lines that extend from the box to the highest and lowest values within 1.5 interquartiles above and below the median, excluding extreme values (open circles up to 95% and asterisks above 95%). A line across the box indicates the median.

All fecundity-related parameters (number of eggs, number of reproductive days, reproductive effort, eggs produced till 10-d old, and age at the first reproduction) were highly related to each other (all correlation values < 0.0001), and not related to longevity (Table 1 and Additional file 2). Thus, a new parameter capable to summarise all parameters associated to fecundity was computed through a Principal Component analysis. The first PC axis explained 92.01% of the total variance. It was directly related to number of eggs, reproductive effort, and eggs produced till 10-d-old, and inversely related to the number of reproductive days and to the age at the first reproduction. This new synthetic parameter was used as a proxy for reproductive fitness. The effect of (1) treatment (D or H), (2) time from start of the experiment (expressed in months and equivalent to the number of desiccations in the D line, called months, M, from now on), and (3) their interaction was tested on fitness components and longevity. A multivariate GLM (Generalised Linear Model) gave significant effects (MANOVA test: all Wilks' Lambda < 0.0001). The desiccation treatment itself was directly related to fitness components (F_1,155 _= 179.52, P < 0.0001), but not to longevity (F_1,155 _= 0.08, P = 0.779). The absence of association between fecundity and longevity may be due to absence of effect, but could be biased by the poor resolution of the statistical test (observed power = 0.059). Months, which represent the time from the start of the experiment, significantly affected longevity (F_2,155 _= 19.89, P < 0.0001), but not fitness components (F_2,155 _= 2.95, P = 0.055). The interaction between treatment (D or H) and months affected both fitness and longevity. Fitness components decreased with time progressively in the H line but not in the D line (Figure 1).

### *Macrotrachela quadricornifera*

Recovery percentages along the 15 desiccation events were 82% – 96% in the first experiment and 88% – 97% in the second one (Additional file 1). Recovery rates did not differ between years (Two-way ANOVA: F_1,78 _= 2.96, P = 0.089), but for each experiment differed between months significantly (F_14,78 _= 2.23, P = 0.014). Apart for the variability, no trend was found in the recovery values.

In the life table experiments, each bdelloid of the D line produced 25 – 27 eggs during the duration of its life, not differently from the initial reference cohort (M0). In the H line the number of eggs produced by each bdelloid was about 20, with a minimum of 15 eggs per lifetime in M8. Reproductive effort remained almost stable through the D line, from M0 (reference) with 1.05 eggs/female x days to M12 with 1.34 eggs/female x days, while it decreased to 0.76 eggs/female x days after 12 months of continuous hydration (Table 1).

**Figure 2:**
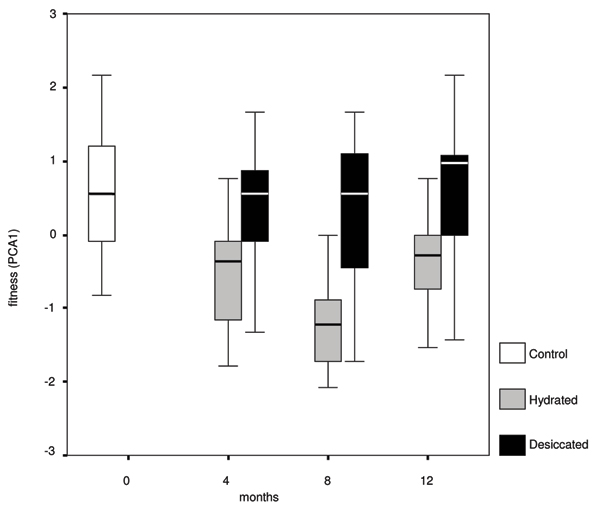
***Macrotrachela quadricornifera*, life history traits**. Box-plot of fitness components at start (control, white) and after 4, 8, 12 months of hydration (gray) and subsequent desiccations (black). The box represents the interquartile range which contains the 50% of values. The whiskers are lines that extend from the box to the highest and lowest values within 1.5 interquartiles above and below the median, which is indicated by a line across the box.

All parameters associated to fecundity (number of eggs, number of reproductive days, reproductive effort, eggs till 10-d-old, and age at the first reproduction) and longevity were closely related (Table 1 and Additional file 3). Therefore, through Principal Component analysis we found a new parameter capable of expressing fecundity, fecundity-related and longevity parameters. The first PC axis explained 99.61% of the total variance and was directly related to number of eggs, number of reproductive days, reproductive effort, eggs produced till 10-d-old and longevity and was inversely related to the age at first reproduction. The effect of (1) treatment (D or H), (2) time from start of the experiment (that is months, M), and (3) their interaction was tested on this single parameter used as a proxy for fitness. A GLM test revealed that fitness was affected by the treatment significantly (F_1,160 _= 105.32, P < 0.0001), by months (M) with a weak effect (F_2,160 _= 9.63, P = 0.055), while the interaction between treatment and months was ineffective (F_2,160 _= 2.04, P = 0.133) (Figure 2).

## Discussion

Both species, like the great majority of bdelloid taxa, are capable of surviving desiccation through anhydrobiosis, although this capacity may differ among the different species [20]. *A. r*. and *M. q*. survive desiccation in high percentages and recover a few hours after the addition of culture medium. The two species, however, came from habitats with different frequencies of drought. *A. r*. was sampled in dry sediments of a billabong, that is fed by floods and may dry out during Australian summer and that represents a temporal water body. *M. q*. was originally collected in a spring-fed-pond that hardly dries out completely. When entering anhydrobiosis, both arrest activity, undergo morphological changes and stop reproduction [21]. At re-hydration, both species resume reproduction ignoring the time spent dry, but while desiccation does not affect lifetime fecundity of *M. q*., it is known to promote post-anhydrobiosis fecundity of *A. r*. [13,15]. In each species a strain was isolated and was desiccated at regular intervals. Due to the relatively short lifespan of the two species, it is unlikely that a rotifer experienced desiccation twice in its life, but this has been found to have no effect on its recovery probability [22]. Recovery rates were mostly invariant during the different months for *A. r*. and randomly variable in *M. q*.; no trend of recovery rates was evident in either species. The fact that a strain after being desiccated reiteratively does not either increase or decrease its recovery capability suggests that neither selection nor adaptation were operating on it. This is not surprising since no genetic variability is expected to occur in a short time under strict ameiotic parthenogenesis.

Nevertheless, in both species 'constant conditions' produced progressive decrease of fitness components with time of cultivation, while the stress induced at regular intervals maintained life-history traits stable in time. How constant a laboratory environment can be throughout years is hard to state, and the condition experienced by a same line in different months may have produced changes of fitness traits. However, paired life table experiments of H and D cohorts were run at the same time under similar conditions: they shared thermostatic chamber, temperature, time of the year, medium, food and experimenters. Yet, the two lines differed, and the responses of the two species to the desiccation were similar, as a general trend. The constantly hydrated line of each species registered a progressive decline in all fitness-related traits, in particular mean life-time fecundity and early reproduction (both age at first reproduction and number of eggs till 10-d-old). In contrast, the strain that was regularly dried had higher fitness-related traits than its parallel H line, but the same traits remained constant if compared to those of the ancestor population, M0. Surprisingly, it is not the treatment that affected fitness, but the absence of treatment that impaired fitness.

It is commonly known in most organisms that processes like reproduction necessitate resources that are diverted from maintenance, with the consequence that high reproduction is associated with short lifespan [23]. Although this does not seem universally true [e.g. [24]], reproduction and fecundity are known to trade-off in monogonont rotifers, as well [e.g. [25]]. However, no inverse association between fecundity and longevity is evident in both bdelloid species; lifespan appears unrelated to fecundity in *A.r*., and is directly, and not inversely, associated in *M.q*. Both species under lab conditions go through a post-reproductive time that may be long; thus, if an early death impedes the rotifer to produce more eggs, the opposite is not true. Surprisingly, recovered bdelloids produce offspring that lay more eggs AND live longer. In other words, the subpopulations of bdelloids that were regularly dried reproduced more and lived longer. But, also maintenance and eventually repair mechanisms are costly processes, so there might be a cost in recovering from desiccation. If this is true, it is not paid in reduction of fecundity or of lifespan.

The differences of fitness correlates between the parallel subpopulations, H and D, must be seen as phenotypic responses induced by the different treatments. We might advance the hypothesis that there could occur some mistake capable of accumulating unless a stressful event reveals its presence by promoting a check-up. Perhaps some 'mistake' reduces fitness but can be removed at recovery after some severe stress, like desiccation. Desiccation implies that the animal loses water, re-adjusting tissues, cells and organules, maybe DNA and ribosomes as well; at re-hydration all structures must resume their original shape and function. But it is not unlikely that structures need to be checked and repaired before activity is resumed. If this is true, then the action can also promote the control over 'damages' accumulated during active life. Thus we can hypothesize that continuous parthenogenetic reproduction, obligatory to the bdelloids, produces the accumulation of some 'mistake' in their cells, and that emergence from anhydrobiosis promotes the removal of the 'mistake' and the re-establishment of the original condition. Alternatively, it could be that bdelloids host viruses or parasites, whose load increases over generations reducing the fitness of the population if conditions are constantly suitable. If conditions become harsher and the virus or the parasite are less tolerant to desiccation than the bdelloids themselves, then the desiccation could represent a way to decrease the parasite/virus load and keep animal fitness constant.

During the duration of the experiment, we observed a regular and progressive decrease of fitness. If this result means that the bdelloid populations maintained permanently hydrated are committed to extinction in the long run, is premature to state. We can imagine that fitness may drop and then level off, possibly at a level sufficient to sustain long-term survival, or, alternatively, the decay of the population can be overcome by a large population size. As already stated, not all bdelloid species live in habitats that dry out, but a minority (less than 10% [12]) occur in permanent water bodies, and few of them were found not to recover after desiccation [20]. Apparently these species do not become extinct in absence of anhydrobiosis. We might advance the hypothesis that these species are all recent ones and are doomed in the long run, but this aspect requires further investigation. On the other hand, based on a previous experience on another bdelloid species [11], even a single event of desiccation improves its fitness components and can thus rescue the hydrated population. On the basis of these observations, it seems unlikely that the decline is due to detrimental mutations, as predicted by current theory [see [26] and literature therein], but it seems more likely to be epigenetic in nature.

## Conclusion

Bdelloids, although aquatic animals, not only are efficient in tolerating desiccation, but are somehow dependent on anhydrobiosis, a circumstance that might represent a key event in their life cycle. If this is true, life in unpredictable habitats should not be seen as the result of competitive exclusion from more 'easy' habitats, but a necessity for long-term survival of a bdelloid rotifer.

## Methods

All experiments were carried out at 24°C. Both species were cultivated in deionized water as a medium and fed with 2–3 mg/ml suspension of powdered fish food (Friskies^®^) [15,19,27].

### Desiccation procedure

Desiccation was performed every 30^th ^day for 15 months and the experiment was replicated in two different years. The whole population was desiccated by transferring the animals on filter paper substrates and by removing excess water by gentle filtration. During each desiccation event, 3–5 batches with about 250 rotifers each were used to test their recovery rates. Anhydrobiosis was induced in a humido-thermostatic chamber according to a previously established desiccation protocol ("C" in [21]). The anhydrobiotic animals were re-hydrated after 7 days of desiccation. Only active bdelloids were considered alive and recovery percentage was recorded about 24 h after water addition. Differences in recovery rates have been tested by ANOVA on original data, as they were normally distributed.

### Life table experiments

Life-table experiments were run at 24°C under continuous dark condition. For each species a first life-table experiment was run before starting the desiccation series and represented the M0 reference. Paired life table experiments of D and H lines were performed after 4, 8, and 12 months, that is after 4, 8, 12 desiccations. At prefixed times, life-table cohort experiments were set up by isolating about 30 newly laid eggs from each D and H population of both species. Along the D line, the eggs isolated were laid soon after recovery from desiccation. The hatched bdelloids represented the experimental cohort and each rotifer received about 5 μg of food per day for the duration of its life. Daily, culture medium and food were renewed, laid eggs were counted and removed and deaths were recorded. From each experiment, a life table was compiled [28]. From the life table data, fecundity (number of eggs per rotifer per lifetime), number of reproductive days, reproductive effort (number of eggs per reproductive days), eggs produced till 10-d-old, age at the first reproduction, and longevity were calculated.

For statistical analysis, life-history parameters have been transformed when data were not normally distributed or resulted in non-normally distributed residuals. Correlation analysis between parameters obtained from life table has been performed with Pearson test, in order to reduce the number of parameters to analyse, avoiding redundancy. This factor reduction has been performed with Principal Component analysis on a covariance matrix.

Uni- or multivariate GLM (Generalised Linear Model [29]) was performed to test the effect of (1) treatment (H or D), (2) months (number of subsequent desiccations in the D lines can reveal a cumulative effect of the desiccation), and (3) interaction between treatment and months.

## Competing interests

The authors declare that they have no competing interests.

## Authors' contributions

All Authors contributed equally to this work.

## Supplementary Material

Additional file 1 **Recovery percentages of *Adineta ricciae *and *Macrotrachela quadricornifera***. Recovery percentages after each monthly desiccation is plotted against time, expressed in months. The results of the two different years are given in different colours; white first year and grey second year. Data outside 1.5 interquartiles are given as open circles. Click here for file

Additional file 2 **Multiple correlation test between life-cycle parameters of *Adineta ricciae***. Pearson correlation values are reported above the diagonal (upper-right part of the matrix); p-values are reported below the diagonal (bottom-left part). Significant correlations are marked by an asterisk. Click here for file

Additional file 3 **Multiple correlation test between life-cycle parameters of *Macrotrachela quadricornifera***. Pearson correlation values are presented above the diagonal (upper-right part of the matrix); p-values are presented below the diagonal (bottom-left part). Significant correlations are marked by an asterisk. Click here for file
