# Supplementary figures and images for: Stress and fitness in parthenogens: is dormancy a key feature for bdelloid rotifers?
Source: BMC Evol Biol. 2007 Aug 16;7(Suppl 2):S9. doi: 10.1186/1471-2148-7-S2-S9 (PMC1963474; doi:10.1186/1471-2148-7-S2-S9)

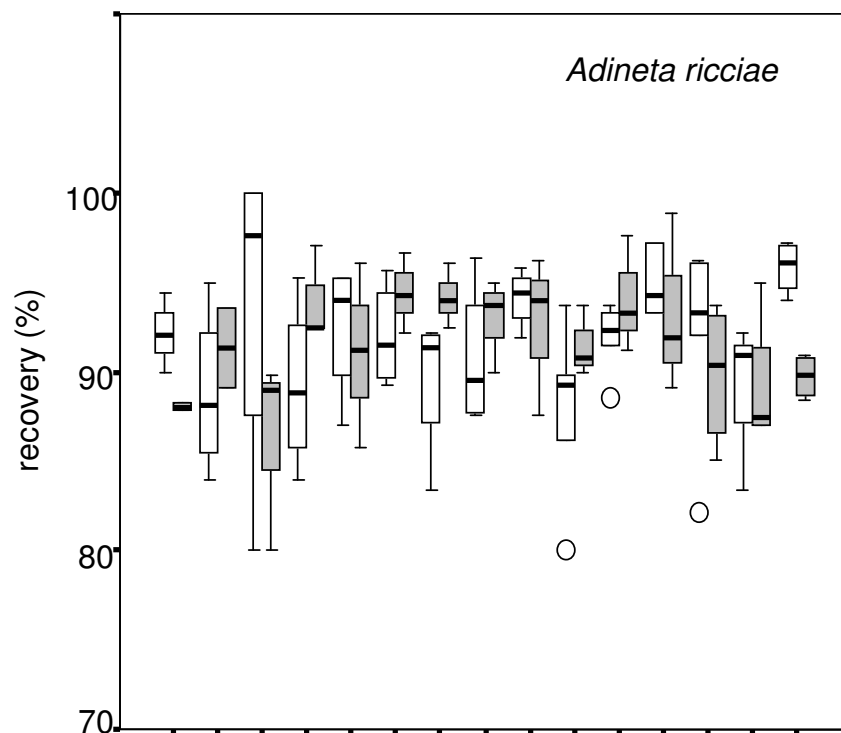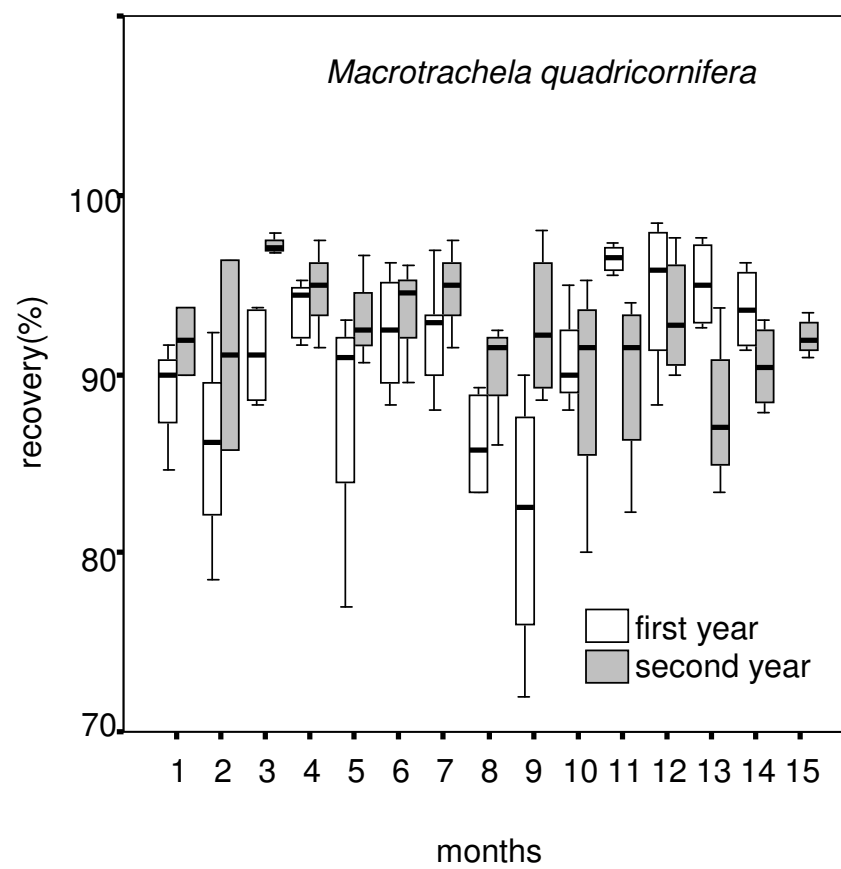

Supplement: Additional file 1 — Recovery percentages of Adineta ricciae and Macrotrachela quadricornifera. Recovery percentages after each monthly desiccation is plotted against time, expressed in months. The results of the two different years are given in different colours; white first year and grey second year. Data outside 1.5 interquartiles are given as open circles. [file 1471-2148-7-S2-S9-S1.pdf]
