# Supplementary material for: Stress and fitness in parthenogens: is dormancy a key feature for bdelloid rotifers?
Source: BMC Evol Biol. 2007 Aug 16;7(Suppl 2):S9. doi: 10.1186/1471-2148-7-S2-S9 (PMC1963474; doi:10.1186/1471-2148-7-S2-S9)
Supplement: Additional file 2 — Multiple correlation test between life-cycle parameters of Adineta ricciae. Pearson correlation values are reported above the diagonal (upper-right part of the matrix); p-values are reported below the diagonal (bottom-left part). Significant correlations are marked by an asterisk. [file 1471-2148-7-S2-S9-S2.doc]

|  | (fecundity)^3 | ln(reproductive days) | reproductive effort | (eggs produced till 10-d-old)^3 | age at first reproduction | longevity |
| --- | --- | --- | --- | --- | --- | --- |
| (fecundity)^3 |  | -0.359 | 0.36 | 0.834 | -0.379 | 0.127 |
| ln(reproductive days) | <0.001* |  | -0.329 | -0.458 | 0.957 | 0.09 |
| reproductive effort | <0.001* | <0.001* |  | 0.67 | -0.374 | -0.089 |
| (eggs produced till 10-d-old)^3 | <0.001* | <0.001* | <0.001* |  | -0.476 | 0.032 |
| age at first reproduction | <0.001* | <0.001* | <0.001* | <0.001* |  | 0.111 |
| longevity | 0.073 | 0.204 | 0.209 | 0.654 | 0.116 |  |
