# Supplementary material for: Stress and fitness in parthenogens: is dormancy a key feature for bdelloid rotifers?
Source: BMC Evol Biol. 2007 Aug 16;7(Suppl 2):S9. doi: 10.1186/1471-2148-7-S2-S9 (PMC1963474; doi:10.1186/1471-2148-7-S2-S9)
Supplement: Additional file 3 — Multiple correlation test between life-cycle parameters of Macrotrachela quadricornifera. Pearson correlation values are presented above the diagonal (upper-right part of the matrix); p-values are presented below the diagonal (bottom-left part). Significant correlations are marked by an asterisk. [file 1471-2148-7-S2-S9-S3.doc]

|  | (fecundity)^2 | reproductive days | ln(reproductive effort) | ln(eggs produced till 10-d-old) | age at first reproduction | longevity |
| --- | --- | --- | --- | --- | --- | --- |
| (fecundity)^2 |  | 0.29 | 0.558 | 0.665 | -0.198 | 0.282 |
| reproductive days | <0.001* |  | -0.577 | -0.066 | 0.004 | 0.659 |
| ln(reproductive effort) | <0.001* | <0.001* |  | 0.699 | -0.223 | -0.329 |
| ln(eggs produced till 10-d-old) | <0.001* | 0.345 | <0.001* |  | -0.51 | -0.094 |
| age at first reproduction | 0.004* | 0.95 | 0.001* | <0.001* |  | 0.121 |
| longevity | <0.001* | <0.001* | <0.001* | 0.179 | 0.083 |  |
